# Coupled whole-body rhythmic entrainment between two chimpanzees

**DOI:** 10.1038/s41598-019-55360-y

**Published:** 2019-12-12

**Authors:** Adriano R. Lameira, Tuomas Eerola, Andrea Ravignani

**Affiliations:** 1 Department of Psychology, University of Warwick, Coventry, UK; 2 School of Psychology and Neuroscience, University of St Andrews, St Andrews, UK; 3 Department of Anthropology, Durham University, Durham, UK; 4 Department of Music, Durham University, Durham, UK; 5 Artificial Intelligence Lab, Vrije Universiteit Brussel, Pleinlaan 2, 1050 Brussels, Belgium; 6 Research Department, Sealcentre Pieterburen, Hoofdstraat 94a, 9968 AG Pieterburen, The Netherlands

**Keywords:** Behavioural methods, Behavioural ecology, Evolutionary ecology, Biological anthropology, Human behaviour, Animal behaviour, Biomechanics

## Abstract

Dance is an icon of human expression. Despite astounding diversity around the world’s cultures and dazzling abundance of reminiscent animal systems, the evolution of dance in the human clade remains obscure. Dance requires individuals to interactively synchronize their whole-body tempo to their partner’s, with near-perfect precision. This capacity is motorically-heavy, engaging multiple neural circuitries, but also dependent on an acute socio-emotional bond between partners. Hitherto, these factors helped explain why no dance forms were present amongst nonhuman primates. Critically, evidence for conjoined full-body rhythmic entrainment in great apes that could help reconstruct possible proto-stages of human dance is still lacking. Here, we report an endogenously-effected case of ritualized dance-like behaviour between two captive chimpanzees – synchronized bipedalism. We submitted video recordings to rigorous time-series analysis and circular statistics. We found that individual step tempo was within the genus’ range of “solo” bipedalism. Between-individual analyses, however, revealed that synchronisation between individuals was non-random, predictable, phase concordant, maintained with instantaneous centi-second precision and jointly regulated, with individuals also taking turns as “pace-makers”. No function was apparent besides the behaviour’s putative positive social affiliation. Our analyses show a first case of spontaneous whole-body entrainment between two ape peers, thus providing tentative empirical evidence for phylogenies of human dance. Human proto-dance, we argue, may have been rooted in mechanisms of social cohesion among small groups that might have granted stress-releasing benefits via gait-synchrony and mutual-touch. An external sound/musical beat may have been initially uninvolved. We discuss dance evolution as driven by ecologically-, socially- and/or culturally-imposed “captivity”.

## Introduction

Dance is one of the richest facets of human expression. A staggering abundance of forms can be found across the world’s cultures (UNESCO Intangible Cultural Heritage List), together with a plethora of mesmerizing analogous performances across the animal world^1–6^. Yet, there seem to exist no parallel behaviours among our closest relatives, the (nonhuman) great apes, calling into question how and why this trait emerged within the terminal branch of our phylogenetic tree and subsequently evolved so expansively^7–9^.

Dance, as a human social event, is propped on *rhythmic entrainment* – the capacity of (at least) two individuals to synchronize with instant precision to a shared and steady rhythm, characteristically manifested through whole-body movement. Whether seen as an evolutionary by-product, or adaptation to promote social cohesion for religion or war, speculation abounds on possible scenarios for the emergence of rhythmic entrainment in the human clade. Despite a burgeoning science of music evolution^10^ and the recent formulation of testable frameworks about the evolution of dance^11^, understanding the proto-stages of dance in the human lineage hinges on what may be learned from primate comparative research and meticulous analyses of rhythm and synchrony^12^. However, the evidence gathered thus far within the primate order does not paint a coherent picture of how dance evolution may have played out^13^. For example, experimental studies with monkeys and great apes have focused on rhythmic synchronization primed by humans under dedicated training-protocols where subjects did not engage with conspecifics^14,15^. In natural or naturalistic conditions, nonhuman primates engage in rhythmic behaviours^16–19^, some of which can be compared, at a purely mechanical level, with the use of music instruments^20,21^. Analyses of these behaviours have been seldom done in light of dance evolution and rarely adopted accurate measures of rhythm and synchrony, with only a few exceptions in the field^22^. Critically, there is a lack of examples in great apes with obvious social and whole-body components that could establish a direct parallel with rhythm entrainment as observed in human dance. Do (or can) our closest relatives engage in dance-like behaviours at all? Only in the presence of such putative examples may evolutionary hypotheses be sensibly formulated about the form and function of dance precursors in the human clade.

**Figure 1:**
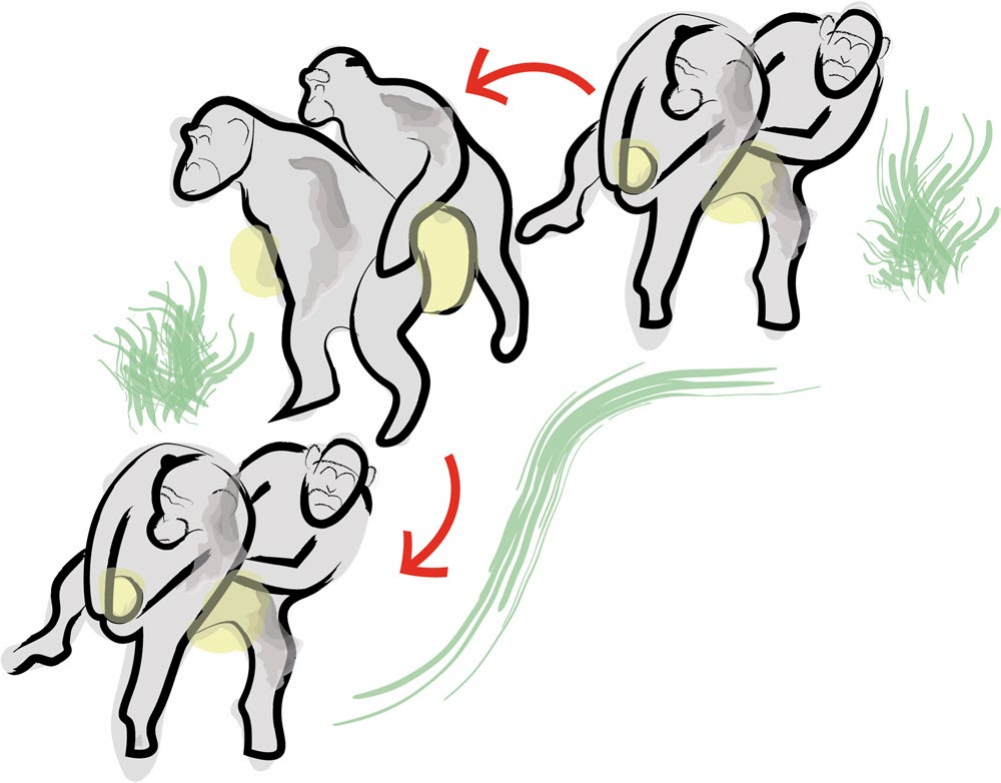
Illustration of the two chimpanzee subjects performing synchronous bipedalism (by ARL).

Here, we present the first evidence for co-synced rhythmic entrainment between two great apes in a naturalistic environment. Holly and Bahkahri, two captive chimpanzees (*Pan troglodytes*) at St Louis Zoo (Missouri, USA), engaged in coupled bipedalism, one following the other in near contact, similarly to a Conga line in humans (Fig. 1, see Supplemental Material for video links).

## Methods

### Study subjects

Chimpanzees were recorded at the Saint Louis Zoological Park, Missouri, United States. Holly (Studbook ID 1064) and Bahkahri (1065) performed the behaviour occupying at all times the same relative position to each other during synchronous bipedalism, with Holly in the front and Bahkahri in the back. The two females have the same age and were both born in 1998 with two weeks apart, although at different zoos. Both were transferred to the St Louis Zoo, with one day apart in that same year, when they were less than 4 months old. The mothers accompanied neither transfer. The behaviour exhibited by Holly and Bahkahri may have developed at the St Louis Zoo, where the two females have lived throughout virtually all their lives, although the exact timing of the behaviour’s inception is uncertain and could precede their arrival at the zoo. At the time of writing, they share the enclosure with 6 other chimpanzees, with an outside area of several thousands of square foot space surrounded by rocky cliffs, offering different climbing structures and composed by vegetation types providing a naturalistic setting similar to the natural habitats where chimpanzees occur in the wild.

The zoo displayed chimpanzee entertainment shows until 1982 when it ceased such performances as an expression of its ethical concerns and priority towards chimpanzee conservation and welfare. The development of the behaviour in Holly and Bahkahri was not, therefore, the result of human training by caretakers or staff as they arrived at the zoo 16 years later after such performances ceased.

### Data collection

As in previous related work^5^, data were collected from videos posted on the global database www.youtube.com. Videos were searched using the terms “conga line,” “chimpanzees,” and/or “St Louis”. Twenty-three videos were found and spanned from October 2011 and April 2015 (see Supplementary Materials). All videos were uploaded at different dates and from different users. Videos were recorded at a resolution of 30 frames/second as indicated by Youtube advanced settings. No videos recorded the same event (e.g. from different angles), which was verified either by the position and direction of the subjects in their enclosure or by the background noise during the recordings. All videos were recorded behind enclosure windows.

These data were publicly recorded opportunistically based on non-continuous observations and were not the result of a video-screening or data-mining exercise by the authors. Accordingly, the number of bouts observed is presumed to represent a far underestimation of the actual frequency of the behaviour. It can be deduced from the available material that individuals engaged in the behaviour at least up to four times in one day (i.e. four bouts of the behaviour were recorded on the same date). We do not describe incidental moments when two chimpanzees moved together and at the same time, but instead a conspicuous behavior.

### Data analysis

Behavioural coding was conducted using Cowlog web version^23^. One of the authors coded the moment of foot-ground contact for each bipedal step for both individuals, setting frame speed to slow motion, namely between 1 and 5 frames/second. Coding was done based on direct observation of foot motion or individuals’ gait at leg and shoulder height. The reliability of the annotations was assessed by having a second coder annotating seven random bouts containing 366 steps. The median difference in onset timings between the annotators was 0.05 s (SD = 0.076 s), and this error was normally distributed (17.9, p < 0.001 in Anderson-Darling test of normality), suggesting that the discrepancies were small, random, and approximately the duration of one video frame (0.0417 s).

The statistical analyses were conducted with R^24^ using the *circular*^25^ and *vars*^26^ packages. For temporal dynamics analysis, the discrete step times were converted into time-series at 100 Hz sampling rate and filtered with third-order non-causal, Butterworth filter for maximal clarity. The annotated timing data (raw data, Fig. S1) is available as Supplementary Material File.

### Ethics statement

This study complied with ethical guidelines and good research conduct for image-only analyses of videos publicly deposited in YouTube. Human subjects were neither involved in this study nor portrayed in any of the analysed videos. No information was used for analyses about any human person, social media user or social media behaviour. This study has in no manner reused or distributed these videos. No copyright or copyright infringement has been intended in this study.

## Results

### Qualitative description of the behaviour

The individual initiating synchronous bipedalism, either front or hind, cued the other through gesture or posture. Occasionally they began rocking their upper body while sitting (commonly out of phase) before starting the behaviour. By the first or second step individuals were in phase and synchronous. Synchrony was maintained even when walking over large obstacles, such as a fallen log, or when making a turn of 180**°**. Synchronous bipedal walk was progressive, i.e., individuals always covered some distance with each step. Individuals typically collected some object before engaging in the behaviour, namely a blanket or bundle of straw. The front individual held objects with both hands, whereas the hind individual used only her right hand while maintaining contact with the front individual with her left hand. Individuals were always within few centimeters from each other. Individuals occasionally paused the behaviour and resumed it, taking with them the same objects in the same original direction. In one occasion individuals performed the behaviour without holding objects and objects seemed available throughout the enclosure, without an obvious function of transport. On this occasion, the front individual presented a quadrupedal gait primarily, while the hind individual made dorsoventral contact with the front individual. No interference from other individuals was observed.

### Quantitative analyses of the behaviour

**Figure 2:**
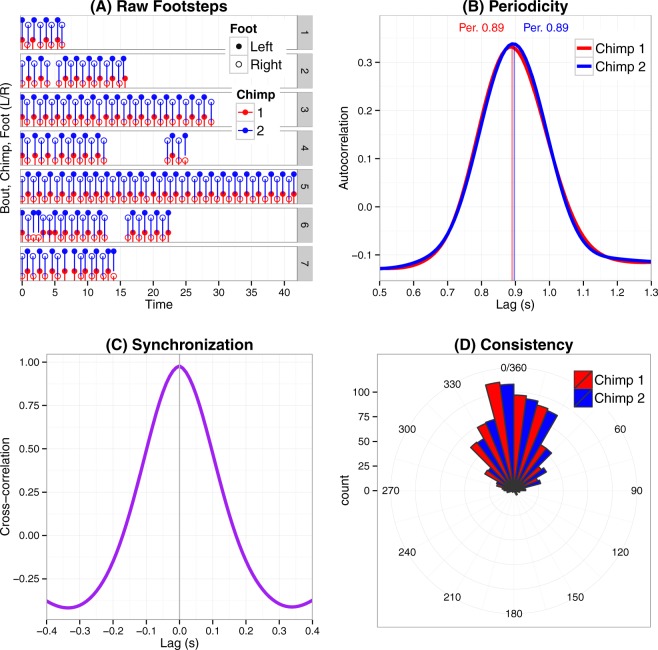
Visualisation of the step data. Panel (A) shows the footstep timings for the first 7 bouts, (**B**) displays the maximal period of both individuals across all bouts using autocorrelation, (**C**) exhibits the synchronization of the individuals using cross-correlation, and (**D**) is the histogram of the step periods when visualized as angles with respect to the prevalent period 0.89 s that equals 0/360 degrees.

For the analysed data, average bout duration of synchronous bipedalism between the two chimpanzees was 24.6 s (minimum = 2.62 s, maximum = 47.84 s, SD = 20.1 s, Fig. S1). Because in various cases the recordings did not encompass the complete behaviour bout, this figure should be taken as a possible lower bound. The two chimpanzees exhibited a mean step duration (tempo) of 0.89 s (Fig. 2A,B), ranging from 0.81 and 1.49 seconds mean tempo per bout (N_bout_ = 28). Standard deviation of step duration for front and hind individuals was 0.154 s and 0.152 s, respectively. This bipedal gait was slower than that recorded in the wild [0.81 s, *t*(391) = 10.56, *p* < 0.001]^27^ and faster than gait tempo in captivity [1.21 s, *t*(391) = −38.84, *p* < 0.001]^28^ in the genus *Pan*. This behaviour was, therefore, overall within the range of typical bipedal walking pace performed by single individuals.

Auto-correlation analysis (focusing on 0.5–1.5 s range) on the time series of individual step patterns yielded a maximal autocorrelation at 0.89 s across all bouts for both chimps (Fig. 2B), but maximum period varied across bouts (median = 0.90 s, SD = 0.055 s, Fig. S2). A cross-correlation analysis (from −0.5 to +0.5 seconds in 1/24 video frame increments) between the time-series from both individuals showed a maximum lag of 0 s across all bouts (Fig. 2C), even though a relative minor variation occurred across bouts at the millisecond scale (mean cross-correlation maximum = 0.0014 s, SD = 0.008 s, Fig. S3). These results indicate that the individuals were constant in tempo.

To investigate the consistency of step periods for both individuals, the periods were analysed concerning the angle of step in reference to the mean step period (period = 360° = 0.89 s, where the first step was set 0° angle) using circular statistics. The mean angle for the individuals was close to 0° (1.7°, SD = 2.5° for front chimpanzee and 1.8°, SD = 2.7° for hind chimpanzee, across all bouts for both individuals, Fig. 2D). Neither of these distributions were uniform (Rayleigh test = 0.78 and 0.77 for front and hind chimpanzee both p < 0.001). The steps expressed as angles across the common period also exhibited a significant correlation (r_JS_ = 0.939, p < 0.001, bootstrapped CI_95_ = 0.905–0.972). These results corroborate that subjects always maintained synchrony even though their exact body cadence exhibited different subtleties.

There was no significant overall difference between the individuals in step period using circular analysis of variance [F_c(1,1292)_ = 0.004, p = 0.95], although the bouts themselves exhibited a significant difference [F_c(27,1266)_ = 9.55, p < 0.001]. These results indicate that the chimpanzees always maintained synchrony regardless their rhythmic pace.

Granger causality analyses with optimal lag (0.28 s, determined by Akaike criterion within vector autoregression across lags 0 to 0.3 s) suggested that the front chimpanzee predicted the hind chimpanzee at the p < 0.05 level in 9 bouts, whereas the reverse was true in 10 bouts. In the remaining 19 bouts there was a two-way causal relation between individuals. Thus, there was no consistent leading role, and both individuals may have interchanged the role of the pacemaker.

Rhythmic synchronization comprised the holding of objects (i.e. straw bundles), but no directed transport or object manipulation was apparent (see Supplemental Material), substantiating the behaviour’s idiosyncrasy.

## Discussion

Time series analysis and circular statistics provide evidence of self-elicited rhythmic entrainment between two chimpanzees involving whole-body engagement. This behaviour has been hitherto undescribed in primates outside human dance, hence potentially shedding new light on the potential primitive forms and functions of proto-dance expression within the human clade. Subjects exhibited a gait movement that was individually regular and mutually synchronized, demonstrating joint rhythm keeping. Whenever one individual accelerated or decelerated her pace, her partner matched her pace. Both individuals alternatively took the role of pace-keeper, but in other occasions the rhythm was kept in a two-way fashion without one of the individuals taking a statistically-identifiable leading role. Rhythmic entrainment was also dynamic between the two chimpanzees. That is, they kept rhythmic entrainment across a range of different tempos and irrespectively of the absolute pace of their gait movement.

Our analyses rule out the possibility that the two chimpanzees engaged in synchronous bipedalism by sheer chance. The simple independent tallying of one individual performing bipedal gait after the other could not have resulted in rhythmic entrainment with instant centisecond precision on a consistent basis across different pace speeds and across years, as reported here.

### Possible ontogenetic origins of the behaviour

Our analyses do not allow determining the ontogenetic origin of this unique social behaviour of rhythmic entrainment. However, it is likely that it emerged as a result of early sensorial and social (maternal) deprivation caused before the individuals moved to their current accredited institution (see Methods). The development of stereotypical movement is known to occur in similar conditions in other primates^29^, including (“institutionalized”) children^30^ (see video link, https://www.youtube.com/watch?v = VCeWr8OFuEs) due to the lack of maternal contact during infancy. Given that stereotypical behaviours emerge spontaneously, this is could be also the case with this unique example of synced co-stereotypy.

Chimpanzees are known to imitate behaviours of zoo visitors, but these cases of imitation involved exogenous (i.e. human primed/prompted) non-rhythmic individual behaviour^31^. A similar human-seeded learning scenario to explain a distinct case of endogenous rhythmic social behaviour would rely on a chain of unlikely events. First, zoo visitors or staff would have to perform a close replica of the reported social rhythmic behaviour (i.e. conga-line) in front of the chimpanzee enclosure. This would open some basic logistical questions. For example, subjects used virtually the complete extension of the outside enclosure to perform the behaviour but the area associated with the visitor windows (where imitation ought to have been learned) was much smaller (and this is also typically the case with enclosures’ “backstage” with staff-only access). Second, demonstration-time would have to be sufficient to assure accurate transmission of behaviour involving two individuals. In light of what has been successfully implemented with do-as-I-do imitation games between humans and apes^32–34^, demonstrations typically require multiple exposures but have never, to our knowledge, involved the simultaneous training of two individuals. If possible, this would at least compound the necessary demonstration time and require a very sophisticated reward method to maintain the two subjects equality engaged in the task. Third, only two individuals would have to acquire the behaviour but none of their conspecifics with whom they share their enclosure. Forth, the two subjects would have to continue exhibiting the behaviour well after the human demonstrators had left the scene. Fifth, animal training programs ceased in the early 1980’s at the zoo. It seems that any activity with requiring an intense training protocol would have not been authorised.

### Possible proximate explanations and their implications

This unique case of rhythmic entrainment in chimpanzees involved close physical contact between two individuals. This multimodal connection between individuals – tactile plus visual – probably facilitated maintaining a shared rhythm. The presence of sustained physical contact and proximity suggests that the individuals may have also drew on neurophysiological benefits where prosocial touch acts as a physiological regulator in presence of stressors, similarly to other pervasive behaviours across primates, such as allogrooming and consolation^35^. This effect could help explain why individuals spontaneously engaged in the behaviour (in the course of several years) in the apparent absence of a visually-identifiable function or external reward.

It has been suggested that during our evolutionary history, humans have developed synchronized behaviours as a means for large-scale social bonding^36^. Any speculative or putative large-scale mechanisms could not have evolved, however, in the absence of functional “small-scale” mechanisms that could have been targeted and selected for in the first place. Chimpanzee rhythmic entrainment indicates that some (social) benefits could have sprung bottom-up, starting at the lowest social level, i.e. between two individuals. Indeed, prosocial behaviour in human dancers (viz. how much they like each other, how they feel toward their group, how much they conform to each other’s opinion) emerges when synchrony is kept between specific individuals but not when synchrony is coordinated unitarily across a larger group^37^. This indicates that, despite humans having evolved cultural means to synchronize the behaviour of large groups (e.g. marches), most physiological effects and benefits extracted from synchrony derive from the synced interactions at a small inter-individual level^37^. Such between-individual effects are already observed in human children, where synchrony enables and increases prosocial behaviour in human infants and children^38,39^. These human data and the case of chimpanzee rhythmic entrainment that we report here suggest, thus, that dance may have originally stemmed from dyadic interactions, instead of having emerged to meet the demands and needs of a large group of human ancestors.

Our findings could provide a bottom-up stepping stone for the “social cohesion hypothesis” for dance origins and evolution. Future agent-based computer models, or analyses of similar bipedal behaviours in other great ape populations (e.g. young chimpanzees in African sanctuaries, see Supplementary Material for video links) could allow examining the hypothesis that group-level behaviour derives from social propagation (e.g. via social learning or affective contagion) of entrainment performed initially between some members within the same group.

### Possible evolutionary implications

Our findings carry implications to current theories of speech and language evolution. It has been proposed that only species capable of vocal (production) learning are capable of beat perception and synchronization^3^. The hypothesis does not recognize great apes as vocal learners, but our analyses demonstrating social entrainment, together with a cumulative body of evidence for vocal (production) learning in great apes^40–45^, indicate that chimpanzees and other great apes ought to be included in the comparative framework at the basis of this hypothesis.

Contra what is generally believed, the present case of endogenous social rhythmic entrainment in chimpanzees suggests that dance may not have originally consisted of a multimodal suite of auditory, visual, and other sensorial behaviours, as it occurs today. Dance could have started off evolutionarily as a mute behaviour. The recruitment of other motor-cognitive capacities and, notably, of a simultaneous synchronous sound could have occurred at subsequent stages of dance evolution.

Due to its repetitive nature and because it involved captive chimpanzees, rhythmic entrainment could be interpreted as a stereotypic behaviour^46^. This possibility raises thought-provoking questions about the socio-ecological inducers for proto-dance behaviours in the human lineage. Compared with the cognitive requirements for the evolution of dance and music, the socio-ecology for the evolution of dance and music has remained virtually ignored. Because stereotypic behaviour in captive animals, including primates, can be associated with confinement and distress^46^, it is conceivable that natural conditions imposing similar effects could have played a role in dance (and perhaps more generally, music) emergence.

For instance, paleo-climate change^47^ shifted food source availability and distribution, putatively driving physiological stress, intra- and intergroup competition, effects that would have been further exacerbated where ancestral hominins were constrained into pockets of remaining habitat or ecological refugia. These conditions could have perhaps caused confinement effects similar to those experienced in captivity. Similarly, if the adoption of a cooperative breeding system^48^ took place along the human lineage, this could have driven individuals to live in “crowded” niches, with non-reproducing and/or post-reproducing individuals remaining in the groups.

These specific scenarios will remain speculative until similar behaviours are identified across great apes (and possible other primates), however, considering that endogenous social rhythmic entrainment is now observed in chimpanzees outside human dance, future hypotheses and models of dance evolution should take into account (in some form and extent) that rhythmic entrainment may have emerged as a stress-coping co-stereotypy under increasing ecologically- and/or socially-induced stress. Other human derived features, such as niche construction^49^ through cumulative culture^50^ could have intensified this “natural captivity” or “self-domestication” effect.

## Conclusion

Evolutionary inferences about human dance based on chimpanzee rhythmic entrainment are limited and should be taken thoughtfully. It is critical to note, however, that our data and interpretations contrast radically with scenarios previously put forward for dance and music evolution^8,51^. Past scenarios have been typically inspired either in modern day human dance/musical expression or in communication systems in further related animal lineages, mostly because these were, at the time, the only living models available. Both (human, and non-primate animal) models misrepresent, however, what may have been the function and form of proto-dance among our hominin ancestors due cultural and biological distance, respectively. Somewhat paradoxically and anachronistically, virtually all dance evolution research has, thus far, tested humans or animals with modern 20^th^ and 21^st^ century music^8^.

The case of chimpanzee endogenous social rhythmic entrainment, together with new data-driven testable frameworks^11^, provides an impulse for the advance of the study of dance evolution based on real and concrete behaviours^51^, including in our closest living relatives. Critically, our findings highlight (*i*) the importance of the social cohesion hypothesis for dance evolution via bottom-up evolutionary mechanisms, (*ii*) the importance of the socio-ecological environment as selective trigger for the evolution of human dance and (*iii*) the importance of stress-coping and social consolation behaviours as precursors for the evolution of human dance.

## Supplementary information

Supplementary Information

## Data Availability

All data needed to evaluate the conclusions in the paper are present in the paper and/or the Supplementary Materials. Additional data related to this paper may be requested from the authors.
