# Supplementary material for: Coupled whole-body rhythmic entrainment between two chimpanzees
Source: Sci Rep. 2019 Dec 12;9:18914. doi: 10.1038/s41598-019-55360-y (PMC6908706; doi:10.1038/s41598-019-55360-y)
Supplement: Supplementary file 1 — Supplementary Information [file 41598_2019_55360_MOESM1_ESM.docx]

**Coupled whole-body rhythmic entrainment between two chimpanzees**

Adriano R. Lameira, Tuomas Eerola, Andrea Ravignani

**Supplementary Information**

**Video links**

List of online video links used as data:

1. Chimp BFFs Doing Conga Line <https://www.youtube.com/watch?v=W9i1RlLcbuI>
2. Chimp Conga Line

<https://www.youtube.com/watch?v=VcvAAfOpiyk>

1. Chimp Conga Line

<https://www.youtube.com/watch?v=DpUZQNMBVrc>

1. Chimp conga line

<https://www.youtube.com/watch?v=fo5pj_oJLw8>

1. Chimp Congo Line at St. Louis Zoo

<https://www.youtube.com/watch?v=0qU9-tJ1_lU>

1. Chimpanzee Congo Line!

<https://www.youtube.com/watch?v=XZDPms39RX0>

1. Chimpanzee St Louis Zoo

<https://www.youtube.com/watch?v=HYAiWkbHyIA>

1. Chimpanzee St Louis Zoo

<https://www.youtube.com/watch?v=SQlR_sLxols>

1. Chimpanzees mimic prominent members of community. Saint Louis Zoo in Missouri (animal socialization)

<https://www.youtube.com/watch?v=5NarwPWptSM>

1. Chimps Syncronized Walk at the St. Louis Zoo

<https://www.youtube.com/watch?v=kN9vLqafa68>

1. Funky Chimp Waddle St. Louis Zoo Feb 2011

<https://www.youtube.com/watch?v=WVO1ltsvOI4>

1. Funny Chimpanzees at Saint Louis Zoo

<https://www.youtube.com/watch?v=hQS02FXGxHs>

1. It's A Love Conga Line

<https://www.youtube.com/watch?v=pKLb-SbOQJQ>

1. St. Louis Zoo Chimps

<https://www.youtube.com/watch?v=f0sgzhSDklw>

1. STL Zoo Chimp March - Part 1

<https://www.youtube.com/watch?v=-WUk-ZfI-ag>

1. Synchronized Chimps Dancing the Conga!

<https://www.youtube.com/watch?v=3pwHD0vKLCo>

1. Synchronized Walking Chimps At St. Louis Zoo

<https://www.youtube.com/watch?v=a0zuOCku16Q>

1. The Chimp Twins

<https://www.youtube.com/watch?v=fEYBkTQLBNQ>

1. Two chimps (staK and Troy) playing-parading at the St. Louis Zoo

<https://www.youtube.com/watch?v=diuHtDYKiUA>

1. Two Chimps Monkeying Around in St.Louis (HD)

<https://www.youtube.com/watch?v=rMIow0Gu_W8>

1. Two funny chimpanzees in St. Louis zoo

<https://www.youtube.com/watch?v=-CWTrfGfLeM>

Video links to similar behavior at Sanctuaries:

1. Chimp Conga Line!

<https://www.youtube.com/watch?v=C39sWYF6P5U>

**Table S1. List of online video links used as data, recording dates and bouts.**

| **Bout** | **Name (YouTube title)** | **Recording Date** | **Link** |
| --- | --- | --- | --- |
| 1 | Chimp BFFs Doing Conga Line | Nov 30, 2014 | W9i1RlLcbuI |
| 2 and 3 | Chimp Conga Line | Jan 27, 2014 | VcvAAfOpiyk |
| 4 | Chimp Conga Line | Dec 15, 2013 | DpUZQNMBVrc |
| 5 | Chimp conga line | Nov 11, 2011 | fo5pj_oJLw8 |
| 6 - 9 | Chimp Congo Line at St. Louis Zoo | Fev 17, 2014 | 0qU9-tJ1_lU |
| 10 | Chimpanzee Congo Line! | Dec 16, 2013 | XZDPms39RX0 |
| 11 | Chimpanzee St Louis Zoo | Aug 15, 2014 | HYAiWkbHyIA |
| 12 | Chimpanzee St Louis Zoo | Jan 18, 2015 | SQlR_sLxols |
| 13 and 14 | Chimpanzees mimic prominent members of community. Saint Louis Zoo in Missouri (animal socialization) | Apr 9, 2014 | 5NarwPWptSM |
| 15 | Chimps Syncronized Walk at the St. Louis Zoo | Oct 11, 2013 | kN9vLqafa68 |
| 16 | Funky Chimp Waddle St. Louis Zoo Feb 2011 | Dec 3, 2013 | WVO1ltsvOI4 |
| 17 | Funny Chimpanzees at Saint Louis Zoo | Jun 1, 2014 | hQS02FXGxHs |
| 18 | It's A Love Conga Line | Fev 17, 2014 | pKLb-SbOQJQ |
| 19 | St. Louis Zoo Chimps | Mar 13, 2014 | f0sgzhSDklw |
| 20 | STL Zoo Chimp March - Part 1 | Dec 19, 2013 | -WUk-ZfI-ag 16. |
| 21 and 22 | Synchronized Chimps Dancing the Conga! | Apr 9, 2015 | 3pwHD0vKLCo |
| 23 | Synchronized Walking Chimps At St. Louis Zoo | Oct 24, 2011 | a0zuOCku16Q |
| 24 | The Chimp Twins | Jun 23, 2012 | fEYBkTQLBNQ |
| 25 | Two chimps (staK and Troy) playing-parading at the St. Louis Zoo | Dec 29, 2013 | diuHtDYKiUA |
| 26 | Two Chimps Monkeying Around in St.Louis (HD) | Dec 19, 2013 | rMIow0Gu_W8 |
| 27 | Two funny chimpanzees in St. Louis zoo | Mar 25, 2015 | -CWTrfGfLeM |
| 28 | * | Oct 31, 2013 |  |

* This video has been removed from the web. This video was part of our data corpus and we have a copy in our depository.

**Fig S1.** Timing of the footsteps across the 28 movement bouts across the two chimps

**Fig S2.** Overall period of footsteps within each individual chimpanzee across the movement bouts using autocorrelation analysis (Chimp 1 = red, Chimp 2 = blue).

**Fig S3.** Graphic representation of average synchrony accuracy (cross-correlation) per bout
